# Empower Peers 4 Careers: Positive Peer Culture to Prepare Adolescents’ Career Choices

**DOI:** 10.3389/fpsyg.2022.806103

**Published:** 2022-05-06

**Authors:** Claudia Schellenberg, Christoph Steinebach, Annette Krauss

**Affiliations:** ^1^Institute for Educational Support for Behaviour, Social-Emotional, and Psychomotor Development, University of Teacher Education in Special Needs, Zurich, Switzerland; ^2^School of Applied Psychology, Institute for Applied Psychology, Zurich University of Applied Sciences, Zurich, Switzerland

**Keywords:** Positive Peer Culture, peer groups, social support, career choice, transition, social-emotional competence, vocational orientation lessons, secondary school

## Abstract

For youth with special needs, where cognitive difficulties, behavioral and psychosocial issues are more common, career choice is particularly challenging. The Positive Peer Culture (PPC) approach uses the resource of peer support to systematically build social–emotional competence. Important key elements are that adolescents feel safe to share their own problems and challenges with others, to overcome difficulties and challenges, to take responsibility for their lives, and to help each other. The Empower Peers 4 Careers Project aims to apply the PPC approach to the context of career choice to promote the development of important competences for the transition from school to work. The pedagogical background of the PPC approach in the setting of career choice, as well as the required learning environments for the peers are presented. The peer group meetings are organized following a defined process through which learning forms social–emotional competence, as well as the class climate can be strengthened. In addition, the role of the moderators of the peer groups – such as class teachers or special education teachers – is examined in more detail and the concept is presented of how they are trained on topics such as resilience promotion and strengths orientation in the context of career choice preparation. The project “Empower Peers 4 Careers” will be cientifically monitored over 2 years using a quasi-experimental control group design, which includes quantitative and qualitative methods. A total of 15 classes of the 8th grade (age group: 14-year-olds) of regular and special schools as well as 10 classes as control classes are participating. The results of the evaluation will not be available until 2023. The article presents the concept with the long-term goals, the implementation and didactics, as well as the hypotheses and the procedure for the evaluation.

## Introduction

The transition from school to work can be challenging: it is a challenging developmental task that involves many new uncertainties in an ever-changing world of work and requires decision-making and adaptability on the part of the individual (Masdonati et al., 2022). In particular, adolescents with behavior experienced as challenging and with psychosocial difficulties face special problems when choosing a career. Problems in the social–emotional area (e.g., internalizing and externalizing problems) occur in about 20% of adolescents (Hölling et al., 2014). They often lack important social as well as emotional competence at this stage of life that are crucial for a successful career entry (Schellenberg et al., 2017; Steinebach and Gharabaghi, 2018). A lack of social–emotional competence can negatively affect the personal and professional development of troubled adolescents in the long term and cause severe health economic burdens (e.g., Greenberg et al., 2003; Ewest et al., 2013).

Interventions targeting young people in transition from school to work increasingly focus on strengthening social–emotional competence. These include self-awareness (understanding one’s emotions, personal goals, and values), self-management (ability to regulate emotions and behaviors), social awareness (ability to empathize and feel compassion), interpersonal skills (competences such as communicating clearly, listening actively), and responsible decision making (knowledge, skills, and attitudes needed to make constructive choices) (Weissberg et al., 2015). These competences are crucial for adolescents to learn productively in class, to succeed in school selection processes, and to enter vocational training and employment (Neuenschwander et al., 2012). Accordingly, social–emotional competence as generic competences represent an important overarching educational goal in “Lehrplan 21” (the overall Swiss curriculum for elementary and secondary school).

For a successful transition from secondary school to upper secondary school, social support from close caregivers (parents, teachers, and peers) plays an important role (Lent et al., 2003). Social support can mitigate the negative impact of difficult starting opportunities resulting from socioeconomic disadvantages or disabilities, promoting social–emotional competence, but also positive expectations and exploration behavior in the school-to-work transition (SWT) (Buhl et al., 2018). Peers become increasingly important as role models in adolescence, and their influence on career choice has been widely demonstrated: peers, for example, play a central role in addressing career aspirations (Eberhard et al., 2009), as well as in the development of social–emotional competence (Greenberg et al., 2003). The project aims to target this source of social support from peers at school to improve transitions from school to work.

The approach of Positive Peer Culture (PPC; Steinebach et al., 2018) makes use of peer relationships by drawing on concepts of humanistic and positive psychology, focusing on the needs and strengths of adolescents to promote social–emotional competence. Establishment of a positive togetherness and mutual appreciation are in the foreground. The basic idea is to help peers in a caring and respectful way and thus strengthen themselves (Opp and Unger, 2006). The project “Empower Peers 4 Careers” aims to apply the PPC approach to the context of career choice to promote the development of important competences for the transition from school to work. Pupils meet regularly every 2 weeks during a lesson to discuss a problem related to their career choice, while teachers take on the role of group facilitators. Group meetings include examining one’s own strengths/weaknesses, identifying a feasible career aspiration, a high degree of frustration tolerance, and personal and social skills in job applications and in contact with companies, e.g., during internships (Häfeli and Schellenberg, 2009; Fröhlich-Gildhoff and Rönnau-Böse, 2020). The concept of PPC is explicitly oriented toward the needs of adolescents: like all people, they have the need to experience themselves as competent, integrated in relationships, but at the same time to be able to live self-determined lives and make decisions independently (cf. also Ryan and Deci, 2000). In particular, youth with disabilities are challenged to learn to take responsibility for their lives. For self-determined decisions, practicing and experiencing generosity by being helpful is additionally crucial (Brendtro et al., 2002; Steinebach et al., 2019).

Other stakeholders in the project include teachers and special education and/or school social work professionals. Due to school inclusion, teachers are increasingly confronted with heterogeneous performance prerequisites, which is often enough experienced as a burden (Sandmeier et al., 2017). It is known that not enough succeeds in supporting all learners according to their prerequisites (Prenzel et al., 2004). New cooperative learning forms such as PPC help to conserve these resources. This is particularly true because targeted attempts are made to use specialists from special education/school social work as facilitators of the peer groups.

In Switzerland, there are hardly any known intervention programs that use the mutual support of peers in the career choice process as a resource. Mentoring programs are an exception. However, such mentorships with young adults who already have experience in education or work and act as role models for young people in the career choice process pursue other goals. In addition, there are peer-based approaches to supporting specific target groups in the career choice process. For example, young people take on a caregiving or support role at home (“Young Careers”). In this case, therefore, a specific target group is involved. In Switzerland, there are single studies on the inclusion of peers in the career choice process. They show positive effects of peer groups on setting realistic goals and finding a suitable solution strategy (Bucher and Bolliger-Salzmann, 2004). The PPC approach was introduced and evaluated in Switzerland as a pilot project in the canton of Lucerne at two secondary schools. It was shown that PPC groups should take place over a sufficiently long period of time to be effective (Sonderegger et al., 2018). The introduction of the PPC approach with peers in career choice classes to promote social and emotional competence of the whole school class is thus innovative and fills an existing gap.

## Pedagogical Frame

### Target Group

The “Empower Peers 4 Careers” approach is intended to strengthen all young people in the transition from school to work. However, young people with psychosocial difficulties and problems in the social–emotional area are likely to benefit particularly from the intervention, as the PPC groups strengthen social and emotional competence, which are especially important for the transition to a career. There is currently little robust data on the prevalence of social–emotional problems in adolescence in Switzerland (Wyl et al., 2017). However, it can be assumed that adolescents from lower school education and special schools (where, in addition to cognitive difficulties, behavioral and psychosocial problems also occur more frequently) have poorer mental health on average than adolescents from school types with a more complex level of requirements (including Lampert et al., 2010; Abel and Keller, 2016). Young people in integrated regular schools or from special schools often face additional difficulties when choosing a career: depending on their special difficulties, they may have to abandon illusions and dream jobs to a greater extent, make compromises in their career choice and deal with setbacks (Eckhart et al., 2010). Adolescents with impairments are also often supported by a large network when choosing a career (e.g., disability insurance, job coaches, and therapists), so self-determination in a contradictory environment is not easy (Schellenberg and Hofmann, 2017). The PPC approach is designed to give adolescents the opportunity to share upcoming difficulties regarding their career choice and to support each other (Steinebach et al., 2018).

### Predictors of Success in the School-to-Work Transition

Several models address influences on a successful SWT process. Depending on the model, a “successful transition” is measured differently, for example, in terms of the time it takes to find an apprenticeship, satisfaction, and working conditions such as job security or wages (Nilsson, 2019). Satisfaction with the job also depends on how well the job is perceived to fit the person’s characteristics (such as personality, interests, and skills) (Eccles and Roesner, 2009; Nägele et al., 2017). Masdonati’s (2010) model highlights the importance of socioeconomic factors that directly influence transition success: these include socioeconomic status, gender, migration, and family background. Thus, certain groups of people have more hurdles to overcome in transition, such as young people with lower educational backgrounds, disability, and little social support. Social support can take various forms such as emotional support, social integration, access to networks, or concrete support in the career choice process (Schultheiss et al., 2001).

Other influencing factors relate to personal factors and thus how someone deals with the challenges in transition (Masdonati, 2010). These include characteristics such as persons’ self-perceptions, the persons’ representation of the world of work, the persons’ mastery of the competences required by a company and the person’s perceptions of social support. Personal protective factors include optimism, high self-efficacy, emotion regulation, as well as communication and problem-solving skills (Bolliger-Salzmann, 2014). Such skills and others are considered social–emotional competence (Schick and Cierpka, 2010), or social and self-competences (Melzer and Al-Diban, 2001; Petermann et al., 2010). More recently, the expression of “career choice readiness” has been described as another important personal competency for a successful transition: this refers to a person’s ability and willingness to successfully deal with specific developmental tasks in their career choice process (Hirschi et al., 2018), and can be fostered through the following elements: future-oriented planning of career choice steps, active exploration (e.g., career explorations), decision-making skills training, information processing support, and reality orientation (Jungo and Egloff, 2015). Pursuing a realistic career aspiration is a key goal that adolescents must pursue for a successful transition (Schellenberg and Hofmann, 2017). Regardless of terminology, promoting personal resources has been shown to be effective with adolescents (Delgrande Jordan and Eichenberger, 2016). For example, promoting social–emotional competence is considered one of the most successful single approaches in resource-based transition support.

### Positive Peer Culture Approach

Originally, the PPC approach was developed in the 1970s in the United States by Vorrath and Brendtro for delinquent youth (Steinebach et al., 2018). The concept is now also used in Germany in various schools and institutions (Opp and Teichmann, 2008). The PPC approach tries to make positive use of the special importance of peers for identity development in adolescence. This is especially true in a developmental stage in which the influence of peers becomes greater. The group has a special significance for the development of a positive identity, because social identity is formed by belonging to a certain group (Tajfel and Turner, 1986). The group influences the individual by imparting norms and teaching behaviors, for example, through model learning. In this way, the group also creates options for actions and development (Brown and Larson, 2009). Peer groups can thus contribute quite decisively to a positive and stable identity and to coping with developmental tasks. The group provides the opportunity to compare external perceptions and expectations with one’s own experience and view of oneself. It also helps to design one’s own actions, to practice one or the other, or to offer support in the implementation of the planned behavior. Group meetings are thus seen as a practice space in which adolescents can develop their own competences (Steinebach et al., 2012).

The person is involved in different environmental systems, which can be distinguished into micro, meso, exo, and macro systems (Bronfenbrenner, 1981). The mission is to shape different developmental environments in such a way that they are helpful for the individual development of the young person. In doing so, however, changes in one system will also bring about changes in another. It is conceivable, for example, that group discussions will not only change the class climate, but also achieve a change in the school climate (Kant-Schaps, 2013). In terms of positive youth development psychology, positive environments allow youth to display their cognitive, emotional, personal, and social skills. In these places, they encounter people who care about them and are authentic and competent. Opportunities are created together to learn, try out, and positively engage (Brendtro and Steinebach, 2012).

In the PPC approach, the focus is on a particular problem or challenge. This includes weaknesses and risks of the person and the environment. However, in the search for a solution, youth also recognize their own physical and psychological resources as well as environmental economic, environmental, and social strengths. It is believed that PPC group discussions promote socio-emotional competence, and thus improves qualities such as interpersonal skills, self-confidence and social awareness (empathy). Mutual helpfulness leads to action in which group members experience themselves as self-efficacious (Vorrath and Brendtro, 2013). Thus, overall resilience is fostered. The experience of being helpful to others promotes self-worth, self-efficacy expectations, and one’s own resilience (e.g., James, 2011). To experience the group situation in this way, it is important that the young people take responsibility for each other, for the counseling process in the group, for finding a solution to the problem as well as for implementing the solution. It is important that the accompanying professionals make it clear that success belongs to the young people. In this sense, the group discussions are a place for important positive experiences. The effects then extend into the culture of the school class as a whole as well as the culture of the school as an organization. All in all, PPC thus promotes individual, social, and organizational resilience (Steinebach and Steinebach, 2013). We define resilience “*as the positive adaptation and sustainable development of a system to respond to short- or longer-term everyday challenges or severe stress. Based on internal system processes and through dealing with the environment, the system defines new reference values and develops required competences, and the ability to cope with future stresses improves*” (Steinebach, 2015, p. 557).

### Positive Peer Culture Approach and Career Choice

The project “Empower Peers 4 Careers” adapts the already established concept of PPC to the context of career choice – a central developmental task of youth. Peers have not been used systematically as a resource in career choice preparation at school, but they hold a lot of potential (Häfeli and Schellenberg, 2009). For example, various steps in the career choice process require social–emotional competence that can be practiced and experienced in the context of groups, since everyone is in the same position (Jungo and Egloff, 2015): (1) Clarify the question, who am I? (2) What is the professional world? (3) “What occupations suit me?” (4) “What career do I choose?” and (5) “How do I apply and prepare for vocational training?” Peers can share their experiences on these steps with each other.

Recognizing and using one’s own resources, like for example understanding one’s emotions, ability to regulate emotions and behaviors, ability to empathize, communicating clearly, and having knowledge needed to make constructive choices are among the objectives in the current curriculum. It is emphasized that the corresponding skills should also be promoted, especially for the later requirements in the working and professional world (Bildungsdirektion des Kantons Zürich, 2017). This is where PPC comes in, in that it supports the further development of social–emotional competence. The goal of vocational orientation in terms of Curriculum 21 is also the promotion of resilience: “*In the multi-year education and career choice process, special attention should be paid to securing and confirming success […] In this way, teachers support constructive development and help students to better cope with stressful life circumstances and setbacks in career choices (resilience)*” (Bildungsdirektion des Kantons Zürich, 2017, p. 495). PPC as an intervention program to promote resilience in young people is therefore particularly well suited in career choice classes and is in line with the objectives of the Swiss curriculum.

**FIGURE 1:**
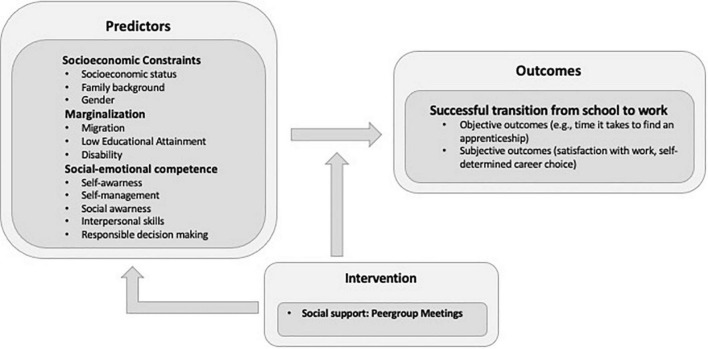
Basic concept of the project “Empower Peers 4 Careers.”

Figure 1 summarizes once again the project’s key areas: social Resources have an impact on success in the school-to-work transition (Masdonati, 2010). At the heart of the Empower Peers 4 Careers intervention is the promotion of social–emotional competence, which takes place through mutual exchange among peers (peergroup-meetings): thus, adolescents are challenged to think about their problems, communicate them, develop dialog and conflict skills as well as emotional regulation skills. All of this helps adolescents make a self-determined, goal-oriented transition (Hirschi et al., 2018; Hofmann and Schellenberg, 2019). By the term successful transitions, we mean successful entry into the post-compulsory school sector, but also satisfaction with the chosen follow-up solution. In Switzerland, after the 9th grade of compulsory education, young people choose either an apprenticeship in a basic vocational training program or a upper-secondary school (“Mittelschule”). About two-thirds of young people enter a basic vocational training program and work 3–4 days in a company and attend 1–2 days of vocational school (Stalder and Nägele, 2011).

## Learning Environment

### Starting With the Needs Orientation of the Young People

**FIGURE 2:**
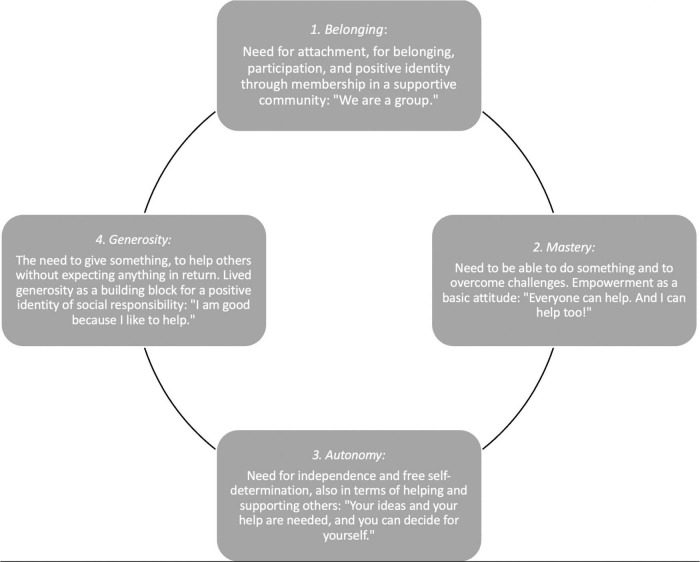
Circle of courage and positive self-development (compare e.g., Steinebach et al., 2018).

Positive Peer Culture is oriented toward various basic needs of adolescents. According to Ryan and Deci (2000), three human needs are initially guiding: experiencing competence, independence, and being included in relationships. Thus, we can assume that group meetings in the PPC approach address these basic needs and bring about activities that lead to the satisfaction of these needs. Thus, the experience of belonging is an important building block for one’s own identity. Furthermore, the independence from the guidelines of others makes it possible to implement one’s own ideas and interests, and the resulting experience of competence ensures self-worth and confidence. And those who have been able to help others will experience themselves as effective and competent. Helping oneself also contributes to the satisfaction of one’s own needs. Thus, in the PPC approach, generosity is understood as another basic need (Brendtro et al., 2002). Figure 2 shows the basic needs of autonomy, mastery, belonging and generosity, which are presented by Brendtro and Larson (2006) in their “Circle of Courage.” Practiced helpfulness is a good way to experience oneself as competent, valuable, confident, and social. The feeling of belonging in a group particularly promotes social–emotional competence in a variety of areas, such as self- and social awareness, and interpersonal skills.

### The Peer Group Meetings

Key elements of the method that contribute to positive adolescent development, according to Vorrath and Brendtro’s (2013) PPC manual, are: (1) Relationships of trust: adolescents feel safe sharing their own problems and challenges with others. (2) Problems as opportunities: overcoming difficulties and challenges builds strength and resilience. (3) Ownership rather than obedience. Young people learn to take responsibility for their lives. (4) A culture of respect. No one hurts another person; everyone is responsible for helping each other. These key elements play an important role in the successful implementation of peer groups and attention should be paid to their observance during meetings.

Peer groups meet regularly with each other to talk about a problem while an adult facilitator is also present. The meetings need clear procedures and group rules to be introduced in the classroom (Table 1). Each meeting begins with each young person naming a current problem that is on his or her mind. In the next step, all group members unanimously agree on who should get the meeting based on the urgency of the problems and the motivation of the individual peers. In the next steps, the group works intensively on the problem, tries to understand it better by asking specific questions and also tries to find out the feelings behind it. Afterward, possible solutions are designed together in order to support the young person as best as possible in overcoming the problem and achieving the goal. Finally, the group leader summarizes the content of the session and, above all, the group process that took place in the round.

**TABLE 1:** Procedure of the group meetings.

Phase of the meeting	Purpose	Aids for career choice
1. Problem appointment	Everyone briefly names a problem.	Prioritize and focus
2. Problem identification	The young people agree on a problem.	Formulating concerns together
3. Description of the problem	The problem is described factually and chronologically.	Formulate or understand issues
4. Asking questions about the situation	Questions are asked about what was not understood.	Differentiate and “explore” the complexities
5. Inquiries about feelings and possible alternative behaviors	Identify problematic behaviors, positive and negative feelings and thought patterns, and new opportunities.	Thinking, feeling, and wanting
6. Design possible solutions	What are the solutions?	Broadening the view and learning to think in scenarios
7. Homework	Who takes on which tasks? What does the group do?	Making agreements, implementing and accompanying solutions
8. Feedback round	The facilitator gives feedback to the group.	Learning from the process

### Implementation of Positive Peer Culture Groups in Vocational Orientation Lessons

The framework in which the group meetings take place is the Career Orientation (CO) lesson. In most schools, career choice preparation is offered across subjects in the last 2 years of school (8th and 9th grade). The groups meet regularly every 2 weeks during a CO lesson (45 min) to discuss a problem related to their career choice. These group meetings are thus built into regular classes and are part of the mandatory program at the high school level (8th and 9th grade). In other contexts, PPC groups are also organized differently, with different duration (up to a maximum of 90 min), and more frequent frequency (up to several times per week, Steinebach et al., 2018). In this project, a compromise was chosen, and the duration and frequency were set according to the possibilities of the curriculum.

Participating schools were given the choice of who would moderate the groups. As a rule, the class teacher is primarily responsible for career choice preparation; in some schools (especially special schools), “a career coordinator” is responsible, or in the integrative setting in the regular school, the school remedial teacher supports the young people (Schellenberg and Hofmann, 2017). The facilitators are trained in advance by the project team on 2 days and familiarized with the PPC approach. On the first day, theoretical knowledge around the implementation of PPC groups is imparted, which they can also practice themselves: they role-play group meetings themselves and each person also practices the role of facilitator. On the second day, the main topic is the introduction of PPC groups at the school, as well as the formation of peer groups among the teachers. The goal is a good networking among the participating adults. After the two training days, the teachers or special education teachers can start with the PPC groups in their classes. Within the framework of additional supervision, they continue to be supported in the implementation of the PPC groups in the classroom (focus: exchange of experiences, What has proven successful? Where are there difficulties?). In this way, the teachers also form a “group” among themselves, report problems to each other and support each other. This could relieve teachers and create free capacities for them. The tasks they take on as moderators also come close to the coaching task called for in the Swiss curriculum, in that they are supposed to adopt “an accompanying and supportive attitude” in career choices (Bildungsdirektion des Kantons Zürich, 2017).

### Tasks of the Moderators

The particular challenge of moderation is, on the one hand, to accompany the groups in such a way that they run in a regulated and successful manner. On the other hand, this must be done in a way that leaves the young people in charge of the meeting and its course (Vorrath and Brendtro, 2013). Only then can the youth attribute subsequent success to themselves. Thus, professionals tend to be facilitators in the discussion sessions and in an observer role. Central to what they do is that they teach values. The highest value is to care about others, to help someone, and to accomplish positive results. The facilitator trusts in the competences of the young people, and promotes the potentials of the young people without dominating themselves. He/she strives to be caring, authentic, empathetic, understanding, sensitive, patient, and confident. Table 2 summarizes key tasks of moderators.

**TABLE 2:** Moderator roles in PPC groups.

**FIGURE 3:**
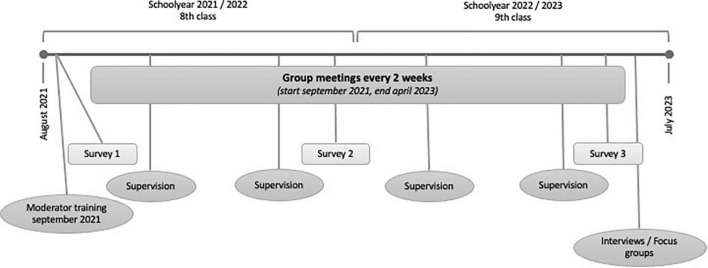
Overview of the methodological design of the project “Empower Peers 4 Careers.”

Tasks of the moderator
The moderator … Ensures adherence to the structure (sequence of steps, cf. Figure 3), forms of interaction and communication, Is not part of the group (shows this by sitting a bit outside the circle), but can intervene at any time, Uses questions as the main means of communication, Does not analyze himself/herself, Does not work out solutions, guides the group to solution-oriented work, Does not use direct forms of address (“you”, “you’re”) and avoids direct dialog and addresses the group (“What does the group think?), Refrains from expressions in the “we” form, avoids “I” messages, Is reserved without appearing apathetic or disinterested, Keeps the flow of communication going, Takes notes for the summary at the end (“feedback round”), Formulates a summary at the end, highlighting positive and helpful behavior, Raises awareness of recognizing and distinguishing positive and negative behavior, Responds to the group’s level of development and passes responsibility.

## Findings to Date, Outlook on the Evaluation

The project “Empower Peers 4 Careers” will be carried out by the University of Teacher Education in Special Needs (HfH) in collaboration with the Zurich University of Applied Sciences (ZHAW) from 2021 to 2023. A total 15 classes of the 8th grade (age group: 14-year-olds) from regular and special schools are participating, which will conduct the PPC groups in class during two school years. The regular school is provided at different levels: in addition to the grammar school level, there is the secondary school level A, which has higher academic requirements than the secondary school level B. In the case of regular schools, it is mainly classes from the less demanding secondary level B that participate; compared to classes from secondary type A, more adolescents with psychosocial (including due to migration background) or social–emotional difficulties are represented, for whom the transition from school to work is more often associated with special difficulties (e.g., Neuenschwander, 2019). Among special schools, those with a focus on social–emotional problems participate. The PPC approach has so far proven its worth especially in this school setting (Opp and Teichmann, 2008; Steinebach and Steinebach, 2009).

Ten additional classes from the aforementioned school types participate as control classes, conducting their usual vocational orientation lessons (without PPC groups). They then have the opportunity to participate in Empower Peers 4 Careers with an 8th grade class in the next school year. This procedure corresponds to a quasi-experimental control group design (Hussy and Jain, 2002), using both quantitative and qualitative data collection methods. Central questions of the scientific monitoring of the project are: How well can the planned group meetings be implemented in the school settings studied? What are the effects on the social–emotional competence of the young people, on their readiness to choose a profession, and on the classroom climate? How can the group meetings be implemented sustainably at the school?

The questions will be examined primarily with the help of standardized measuring instruments, with the written survey of the students taking place at three measurement points. The “Student Assessment List for Social and Learning Behavior” (Schülereinschätzliste für Sozial- und Lernverhalten, SSL) by Petermann and Petermann (2014) will be used to measure social competence. Career readiness is assessed with the Career Resources Questionnaire for Adolescents by Marciniak et al. (2019). For the investigation of class and school climate, the Linz Questionnaire on School and Class Climate on Community, Rivalry, Willingness to Learn, Disruptiveness (Eder, 1998) is used. Further questions concern the development of social–emotional competence (Strengths and Difficulties Questionnaire, SDQ; Klasen et al., 2003). Further, group moderators will be asked, through in-depth interviews, what aspects were important to them in the implementation of Empower Peers 4 Careers. Interviews will also be conducted with selected students, which will serve to selectively enrich the quantitative survey. Figure 3 provides an overview of the most important stages of the entire project.

## Hypotheses on the Effectiveness of “Empower Peers 4 Careers”

The results of our study will be available in 2023. The following hypotheses can already be formulated: the introduction of PPC at school will strengthen social–emotional competence. These are important competences that have been given special weight in the Swiss curriculum “Curriculum 21” and are important for the promotion of resilience and the successful transition to a career. It is expected that the intervention will promote career readiness and thus the ability to choose a realistic, suitable profession and to trust in one’s own abilities even in the face of setbacks. This strengthens the overall career choice process at school, which has a positive impact on finding an apprenticeship in post-compulsory education. These hypotheses are formulated based on findings from other studies that found changes in participating youth in a variety of areas, such as self-awareness and interpersonal skills (Brendtro and Caslor, 2019), academic achievement (Brendtro et al., 2002), school climate (Mitchell and Cockrum, 1980), and decreases in behavior problems (Gottfredson, 1987).

The relatively long intervention period (over two school years with) will in all likely strengthen and stabilize positive changes among adolescents. For example, various authors conclude that positive effects can be expected 12 months after the introduction of PPC at the earliest (Caslor, 2003). Other important factors for success are that PPC is well implemented at the school and that the facilitators are well trained or prepared (Gold and Osgood, 1992).

The focus of the intervention is on young people with problems in the social–emotional area, involving both regular and special schools. Thus, it has learners with different impairments, in the field of ADHD, behavior, autism or with mental health problems. We expect overall positive effects on aspects of emotional experience and behavior (cf. SDQ scale, Klasen et al., 2003) based on relevant literature (e.g., Opp and Teichmann, 2008). However, we assume that the PPC intervention is not equally effective for all target groups. For example, the literature (Ryan, 2006) shows that adolescents with trauma have more relapses into old behavior patterns after a PPC intervention than adolescents without trauma. The authors explain it because traumatized adolescents are less able to trust other people (peers and adults) because of their stressful experiences. This is where the important role of the moderator of the groups comes into play by paying attention to the special needs of all group members.

## Discussion

### Vision and Sustainability

The aim of the project is to support young people in the transition from school to work, paying particular attention to inclusive educational pathways. Career choice should not be viewed as a one-time event, but rather embedded in the context of lifelong learning. In today’s careers, changes are increasingly frequent and an active career path design is necessary to adjust the chosen direction with a view to different life roles. Career choice should also already be understood as part of a holistic “life design” when accompanying young people from school to work (Savickas, 2012).

The project is particularly aimed at strengthening adolescents in der regular school and special schools for behavioral problems. In particular, this is intended to strengthen young people who, because of their preconditions (e.g., in terms of socio-demographic background, school background), have fewer good chances for direct transfer (Hupka-Brunner and Wohlgemuth, 2014). Ultimately, however, the project is intended to support all young people in the school-to-work transition so that the goal of the Swiss Conference of Education Directors (EDK) of raising the graduation rate in the post-compulsory sector to 95% is achieved. The focus of the intervention is on strengthening the social and emotional resources that are important for a successful transition. Social competences, especially communication skills and contact and teamwork skills, have been identified several times in the literature as success factors for at-risk youth in the school-to-work transition (Häfeli and Schellenberg, 2009). By implementing PPC in career choice education, social support from peers is used as a resource in the career choice process. The positive peer approaches can be particularly effective in promoting social competences as well as emotional competences (such as emotion regulation skills or resources to cope with current tasks within the career domain). PPCs offer adolescents the opportunity to satisfy key basic needs of experiencing competence (by taking responsibility for each other), autonomy (through a sense of personal strength), and belonging (through caring, reciprocal interactions), but beyond that, generosity. This contributes decisively to the young people’s self-esteem (cf. Brendtro and Caslor, 2019), building self-efficacy (increasing confidence in one’s own abilities), exercising self-determined career choices (learn to take responsibility for their lives) (cf. also Ryan and Deci, 2000).

The longer-term vision of the project is to strive for a more conducive culture of learning and conversation in schools, with more emphasis on mutual social support among youth. While the pedagogical professionals learn to hand over responsibility to the young people and grant them participation in their own development, the young people learn to help each other and thus contribute to a community with a lived culture of mutual support and solidarity.

The Empower Peers 4 Careers Project will be scientifically monitored to study the effectiveness of the approach for a successful transition to the world of work. An important goal of the planned intervention study is to develop an offer for all cantons and different types of schools so that all young people in the school-to-work transition can benefit from it. It is also important to examine how the approach can be transferred to other types of schools such as 10th grade or bridge programs. It is also conceivable that the PPC approach could also be applicable in the training profession, with work colleagues supporting each other (Valero and Hirschi, 2019). Concrete products for practical use, such as brochures and guidelines, should contain ideas for content, didactic-curricular and organizational implementation with reference to the curriculum.

### Assessments of Success and Feasibility

The project will be carried out during the period of 3 years thanks to the financial support of various funders. In addition, an expert committee consisting of representatives of vocational guidance, the cantons participating in the project, the universities, the elementary school education and of experts of content-related offers accompanies this project.

The training of the moderators of the participating 15 classes has already been successfully carried out. Central for the further success is that the participating persons continue to be well supported by the project team, especially when problems arise. Therefore, the introduction of such an offer should be understood as a school development process (Steinebach et al., 2018). From there, it should also be the goal to anchor PPC with its values, goals, and methods in the school as an organization.

Possible risks for the implementation of the project are that the teachers who have received the training as facilitators leave the school. In this case, the knowledge must be passed on to the new teachers, or the PPC groups must already be so well established that they can continue to run well. Other risks lie in the data quality of the data collected. The written surveys in the classes at the first time of measurement have already taken place and were conducted within one lesson in the class. For meaningful evaluations a sufficiently large sample must be achieved. To achieve this, additional classes could be included in the project for the 2022/23 school year. In addition, the effects of the current pandemic must always be closely monitored, and adjustments may need to be made.

## Data Availability Statement

The original contributions presented in the study are included in the article, further inquiries can be directed to the corresponding author.

## Ethics Statement

Ethical review and approval was not required for the study on human participants in accordance with the local legislation and institutional requirements. Written informed consent to participate in this study was provided by the participants’ legal guardian/next of kin.

## Author Contributions

CSc: project management of Empower Peers 4 Careers, working on the text and illustrations, final editing, and input. CSt: leading the pedagogical training of the facilitators of PPC, in the scientific monitoring of the project, working on the text, translation, and illustrations. AK: project management of Empower Peers 4 Careers, working on the text and illustrations. All authors contributed to the article and approved the submitted version.

## Conflict of Interest

The authors declare that the research was conducted in the absence of any commercial or financial relationships that could be construed as a potential conflict of interest.

## Publisher’s Note

All claims expressed in this article are solely those of the authors and do not necessarily represent those of their affiliated organizations, or those of the publisher, the editors and the reviewers. Any product that may be evaluated in this article, or claim that may be made by its manufacturer, is not guaranteed or endorsed by the publisher.
